# Supplementary material for: Effects of Motilin Receptor Agonists and Ghrelin in Human motilin receptor Transgenic Mice
Source: Int J Mol Sci. 2019 Mar 27;20(7):1521. doi: 10.3390/ijms20071521 (PMC6479874; doi:10.3390/ijms20071521)
Supplement: Supplementary file 1 [file ijms-20-01521-s001.pdf]

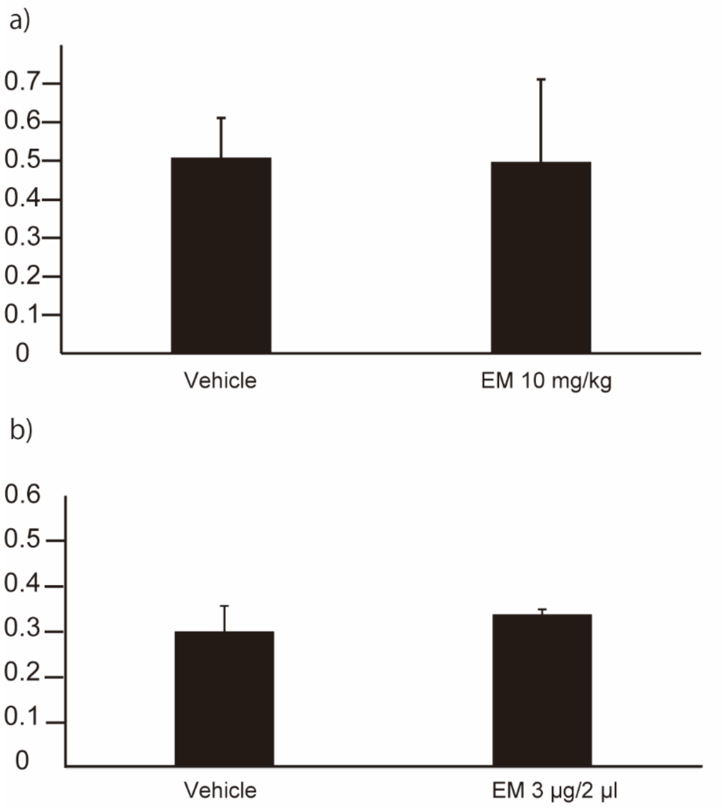

Supplementary Figure S1. Gastric emptying of wild-type mice following peripheral (intraperitoneal) administration of EM (n = 3) (a), or following central (intracerebroventricular) administration of EM (n = 3) (b). Values represent the mean±SD for the indicated number of animals.

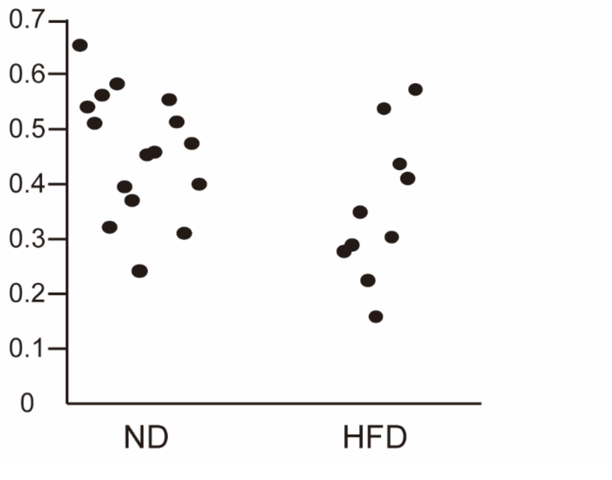

Supplementary Figure S2. Gastic emptying of human *MLNR* Tg mice following administration of ND (normal diet) and HFD (high-fat-diet). Each point represents 1 animal.

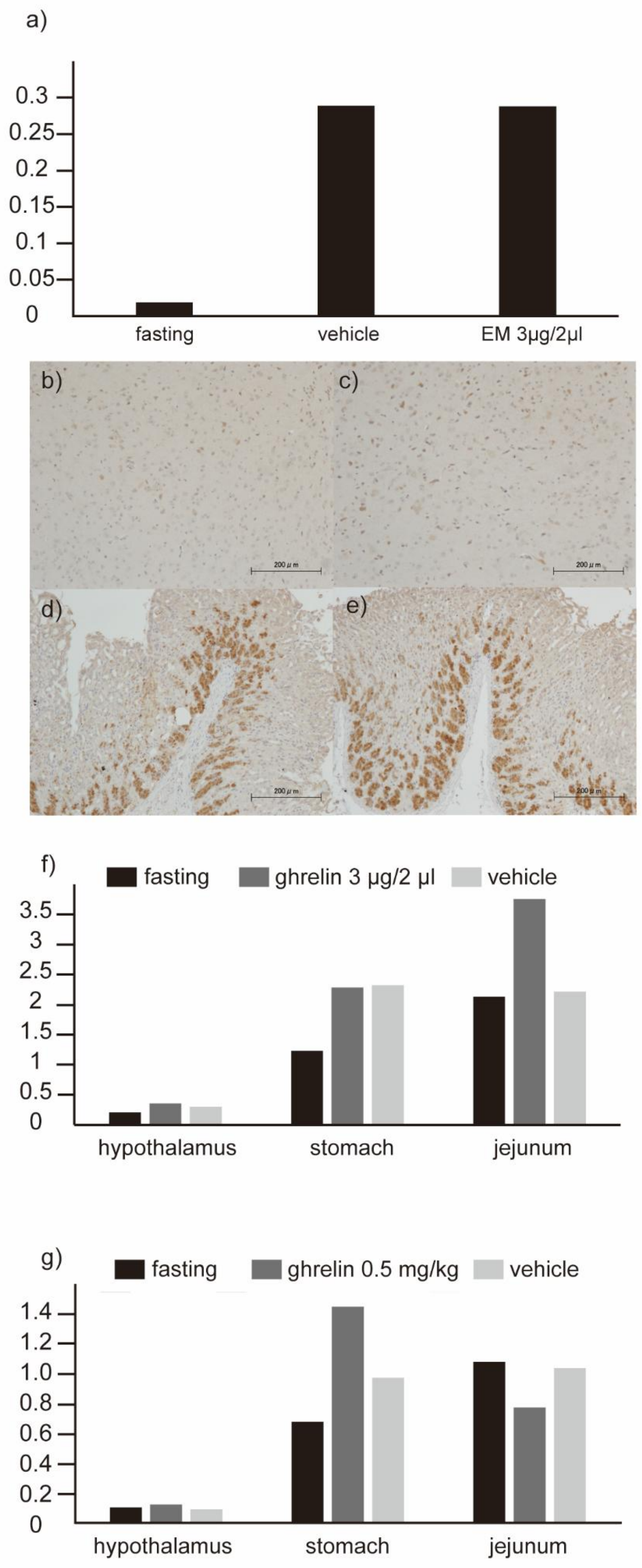
a)
0.3
0.25
0.2
0.15
0.1
0.05
0
fasting
vehicle
EM 3μg/2μl
b)
c)
d)
e)
f)
fasting
ghrelin 3 μg/2 μl
vehicle
3.5
3
2.5
2
1.5
1
0.5
0
hypothalamus
stomach
jejunum
g)
fasting
ghrelin 0.5 mg/kg
vehicle
1.4
1.2
1.0
0.8
0.6
0.4
0.2
0
hypothalamus
stomach
jejunum

Supplementary Figure S3. Accumulation of *GHSR* transcripts in hypothalamus of human *MLNR* Tg mice following central (intracerebroventricular) administration of EM (n = 3) (a). Immunohistochemical staining for GHSR expression. Representive micrographs are provided showing the distribution of GHSR-positive cells (brown staining) in the brain cortex of mice administered vehicle (b) or EM (c), and in the stomach of mice administered vehicle (d) or EM (e). Accumulation of *MLNR* transcripts in hypothalamus, stomach, and jejunum in human *MLNR* Tg mice in fasting (n = 3) and 1 hour following central (intracerebroventicular) administration of ghrelin (n = 3) (f), or following peripheral (intraperitoneal) administration of ghrelin (n = 3) (g). The values shown are the numbers for real-time RT-PCR products of *GHSR* and *MLNR* mRNAs normalized to those of *GAPDH*.
